# Decoding nuclear-encoded mitochondrial genes in major depressive disorder: A multi-omics perspective

**DOI:** 10.1017/S0033291725102559

**Published:** 2025-11-18

**Authors:** Jing Liao, Xianyan Wang, Gaokun Dai, Huilei Xu, Fuchao Zhang, Xiang Yuan, Qiuxia Feng

**Affiliations:** 1 Department of Child and Adolescent Psychology, Nanchong Psychosomatic Hospital, Nanchong, Sichuan, China; 2 Department of Pain Medicine, Affiliated Hospital of North Sichuan Medical College, Nanchong, Sichuan, China; 3 Department of Severe Mental Disorders, Nanchong Psychosomatic Hospital, Nanchong, Sichuan, China; 4 Department of Psychosomatic Medicine, Nanchong Psychosomatic Hospital, Nanchong, Sichuan, China; 5 Outpatient Department, Nanchong Psychosomatic Hospital, Nanchong, Sichuan, China

**Keywords:** GWAS, major depressive disorder, mitochondrial genes, summary-based Mendelian randomization

## Abstract

**Background:**

Mitochondrial dysfunction has been implicated in the pathogenesis of major depressive disorder (MDD); however, the causal contributions of specific mitochondrial genes across regulatory layers remain unclear.

**Methods:**

We integrated genome-wide association study summary statistics from the Psychiatric Genomics Consortium and FinnGen with quantitative-trait-locus (QTL) datasets for DNA methylation, gene expression (eQTL), and protein abundance. Mitochondrial genes were annotated using the MitoCarta3.0 database. Summary-based Mendelian randomization and Bayesian colocalization were applied to assess causal relationships, with colocalization determined by the posterior probability of a shared causal variant (PPH4), and the false discovery rate used for multiple-testing correction. Brain-specific effects were evaluated using Genotype-Tissue Expression eQTL data. Prioritized genes were ranked based on cross-omics consistency and replication evidence.

**Results:**

Five mitochondrial genes were prioritized. *TDRKH* showed consistent associations across methylation, transcription, and protein levels, with hypermethylation at cg24503712 linked to reduced expression and a lower risk of MDD (Tier 1). *METAP1D* (Tier 2) demonstrated protective effects at both the transcript and protein levels. *LONP1*, *FIS1*, and *SCP2* (Tier 3) exhibited consistent but complex regulatory patterns. Several signals were replicated in brain tissues, including *TDRKH* in the caudate and *METAP1D* in the cortex.

**Conclusions:**

This study provides multi-omics evidence for the causal involvement of mitochondrial genes in MDD. *TDRKH* and *METAP1D* emerged as key candidates, offering promising targets for future mechanistic research and therapeutic development.

## Introduction

Major depressive disorder (MDD) is a complex psychiatric disorder affecting more than 300 million people worldwide and imposing major socioeconomic burdens; its multifactorial etiology remains incompletely understood, complicating diagnosis and treatment (Trivedi, 2020). Genetic studies demonstrate a heritable component in MDD, with heritability estimated at 30–50% (Kendall et al., 2021). Mitochondria are increasingly implicated in MDD, not only via bioenergetics but also through oxidative stress, cell survival, inflammation, and immunity processes linked to depression (Büttiker et al., 2022; Chan, 2020; Ciubuc-Batcu, Stapelberg, Headrick, & Renshaw, 2024; Larrea et al., 2024; Lee, Zamudio-Ochoa et al., 2023; Liu et al., 2024; Ye et al., 2025; Zong et al., 2024).

Prior research has examined mitochondria and MDD at selected molecular levels. For example, proteomic studies have reported abnormalities in mitochondrial components within neuronal extracellular vesicles in patient plasma (Goetzl et al., 2021); peripheral immune-cell studies have shown increased markers of mitochondrial fission, mitophagy, and apoptosis (Scaini et al., 2022); transcriptomic analyses coupled with machine learning have proposed mitochondria-related gene signatures with potential diagnostic value (Chen, Tang, Gu, & Zou, 2025; Lei, Chen, Wang, & Zhang, 2025; Liu, Wu, & Li, 2024). Genetic work has linked specific mtDNA variants to MDD in certain subgroups or families (He et al., 2025; Yin et al., 2026) and conventional two-sample MR has suggested possible bidirectional causal relationships between mitochondrial proteins and MDD (Sun et al., 2026). However, these lines of evidence are often limited by single-omics designs, modest sample sizes, and restricted population coverage; a predominance of peripheral tissues with little integration of brain data; limited causal inference without robust colocalization testing; sparse cross-cohort and cross-tissue replication; and an incomplete, systematic evaluation of nuclear-encoded mitochondrial genes. As a result, findings have been difficult to reconcile into a clear and reproducible pathogenic model.

To address these gaps, we developed a larger-scale, methodologically strengthened framework. We integrate two independent European MDD genome-wide association study (GWAS) resources with three classes of molecular QTL – mQTL (epigenetic regulation), eQTL (transcriptional regulation), and pQTL (protein abundance) – to trace the regulatory cascade for the same gene from DNA methylation through gene expression to protein levels (Giambartolomei et al., 2014; Wu et al., 2018). Our core analysis uses summary-based Mendelian randomization (SMR), which treats the cis-QTL as an instrumental variable to mitigate environmental confounding and reverse causation; we pair this with the HEIDI test and Bayesian colocalization to determine whether the molecular signal and disease association share a single causal variant, thereby reducing linkage disequilibrium (LD)-driven false positives (Giambartolomei et al., 2014; Zhu et al., 2016). Unlike conventional two-sample MR, SMR is well suited for high-throughput gene-level screening of QTL–disease relationships when valid instruments are few but effects are concentrated. To ensure reproducibility and biological relevance, we cross-validate between the Psychiatric Genomics Consortium (PGC) and FinnGen and extend analyses from peripheral blood to tissue-specific QTL across multiple brain regions, retaining clinical translatability while directly probing CNS pathology. Distinct from prior work that often focused on mtDNA, we systematically target nuclear-encoded mitochondrial genes curated in MitoCarta3.0, which directly regulate mitochondrial function, have stronger genetic instruments, and present more tractable therapeutic targets (Rath et al., 2021). This design consolidates previously fragmented, single-layer signals into a coherent, reproducible, and mechanistically interpretable chain of evidence.

In sum, by integrating multi-omics with strengthened causal inference, cross-cohort replication, and cross-tissue analyses including multiple brain regions, we identify mitochondrial genes whose regulation is linked to MDD. This framework addresses limitations of prior work, including small mtDNA studies, weak causal validation, single-omics bias, and limited brain relevance, and highlights translational candidates.

## Methods

Large-scale genome-wide association summary statistics for depression were obtained from two independent European cohorts, the PGC and the FinnGen study, together comprising more than 800,000 individuals. These datasets were analyzed alongside quantitative-trait-locus (QTL) resources describing genetic influences on DNA methylation, gene expression, and protein abundance, which together served as the molecular exposures in our framework.

We applied SMR to test causal effects of mitochondrial regulation on depression. We applied quality control to select the strongest cis-QTLs and exclude inconsistent variants. Associations that passed these steps were further evaluated with Bayesian colocalization analysis to determine whether the same genetic variant underlies both the molecular trait and depression outcome.

**Figure 1.**
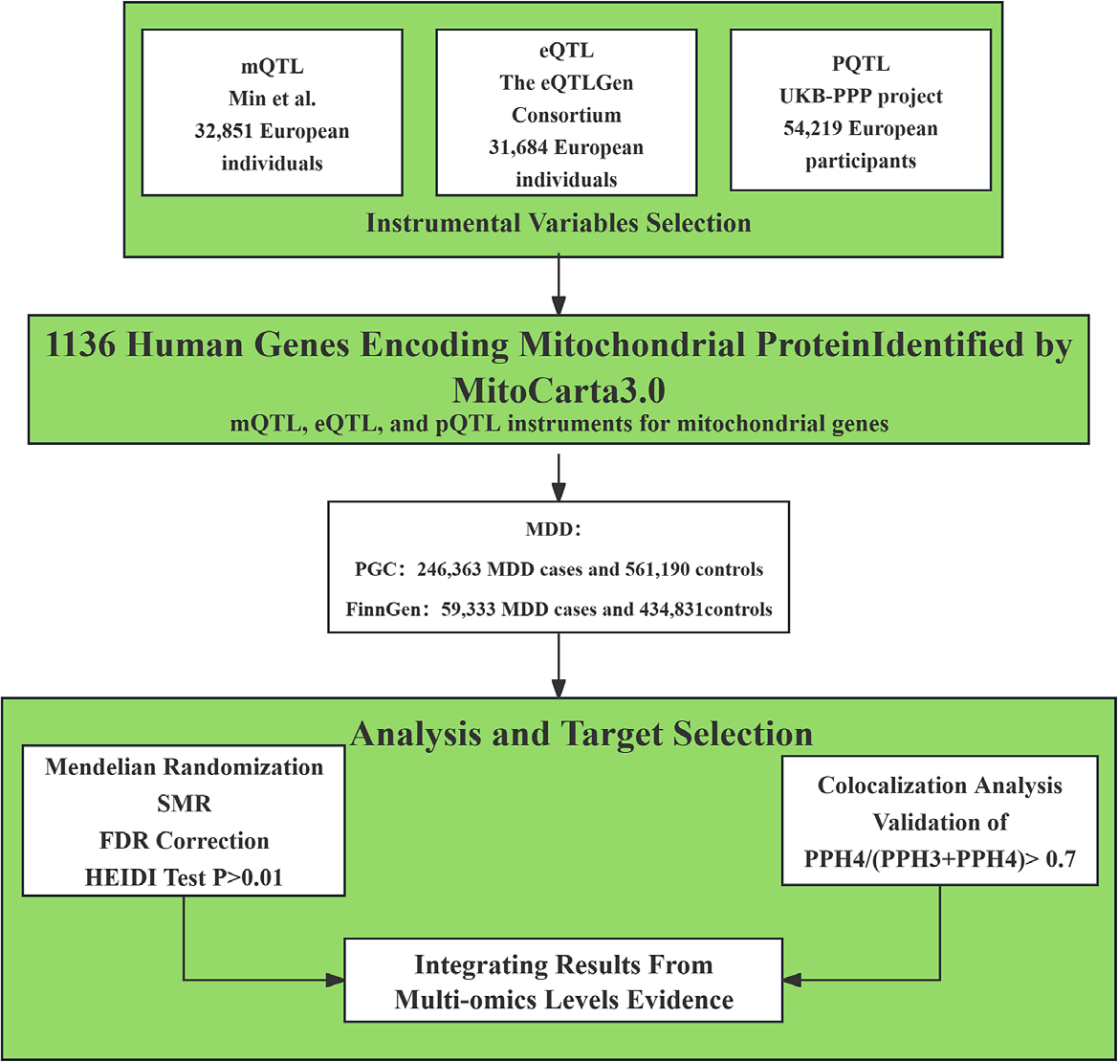
Study design. SMR, summary-based Mendelian randomization; QTL, quantitative trait loci; MDD, major depressive disorder; PPH4, Posterior Probability of Causal Variant.

By combining evidence across methylation, transcription, and protein levels, and by validating findings across independent datasets, we prioritized mitochondrial genes with consistent multilayer support. This integrative approach provided a systematic framework to identify high-confidence candidate genes and to clarify the contribution of mitochondrial regulation to the pathogenesis of MDD. The workflow is illustrated in Figure 1. Ethical approval for the study was obtained from the relevant review board (Howard et al., 2019; Kurki et al., 2023).

### Source of data for methylation, expression, and protein quantitative trait loci

For DNA methylation, we used data from a large-scale meta-analysis conducted by Min and colleagues, which included more than 32,000 participants across 36 population- and disease-based cohorts. Methylation was profiled by high-density arrays with functional normalization and extensive QC (age, sex, smoking, batch, cell composition, and principal components). These measures improved the accuracy and reproducibility of the methylation–genotype associations used in the analysis (Min et al., 2021). Rather than reestimating these associations, we employed the reported mQTL signals as instrumental variables in our Mendelian randomization analyses to infer potential causal pathways linking genetic variants, DNA methylation, and the risk of MDD.

Gene expression data were derived from the eQTLGen consortium, which analyzed whole-blood samples from more than 31,000 individuals. We focused on cis-eQTLs for 19,250 genes expressed in blood. All SNP–gene pairs that met the criteria – namely, a physical distance of less than 1 Mb between the SNP and the gene’s transcription start site and being tested in at least two independent cohorts – were included in our analysis. This rigorous selection strategy ensured both the reliability and biological relevance of the data (Võsa et al., 2021). To investigate potential brain-specific regulatory mechanisms related to MDD, we incorporated brain region-specific cis-eQTL summary statistics from the Genotype-Tissue Expression (GTEx) Project (Carithers et al., 2015; GTEx Consortium, 2020). This resource systematically identified cis-eQTLs derived from whole-genome and RNA sequencing performed on post-mortem samples from approximately 1,000 donors. Our study utilized eQTL data from the following 12 brain regions implicated in mood regulation: amygdala, anterior cingulate cortex (BA24), caudate (basal ganglia), cerebellar hemisphere, cerebellum, cortex, frontal cortex (BA9), hippocampus, hypothalamus, nucleus accumbens (basal ganglia), putamen (basal ganglia), and substantia nigra. In subsequent SMR analyses, GTEx brain eQTLs served as the exposures, with the PGC and FinnGen MDD GWAS datasets serving as the outcomes.

For protein abundance, we incorporated data from the UK Biobank Pharma Proteomics Project. This initiative profiled the plasma proteome in more than 54,000 individuals and performed a comprehensive association analysis across 2,923 circulating proteins. It identified over 14,000 significant genetic associations that characterize the heritable architecture of protein expression (Sun et al., 2023). In the original QTL studies, multiple testing was controlled using the false discovery rate (FDR, *p* < 0.05, Benjamini–Hochberg method), and only associations that passed this threshold were included in the present analyses.

Mitochondrial genes were defined using the MitoCarta3.0 database, a manually curated and experimentally validated catalogue of 1,136 human mitochondrial proteins. It served as the reference standard for selecting mitochondria-associated loci for downstream analyses (Rath et al., 2021).

### Outcome dataset

The discovery dataset was obtained from the PGC, comprising 246,363 individuals diagnosed with MDD and 561,190 ancestry-matched controls. Diagnostic status was established based on self-reported clinical history or treatment records. These data represent one of the largest and most comprehensive resources available for validating depression-related genetic associations (Howard et al., 2019). To validate our findings, we used a replication dataset from the FinnGen study, which comprised 59,333 cases defined using clinical diagnosis codes derived from national hospital discharge records and death certificates, paired with 434,831 population-matched controls derived from Finnish biobank infrastructure (Kurki et al., 2023). For further information, see Supplementary Table S1.

### SMR analysis

We used SMR to test whether genetically regulated mitochondrial methylation, expression, or protein abundance influences MDD risk. For eQTL, mQTL, and pQTL, we used publicly available summary-level association statistics (effect sizes, standard errors, and *p* values), rather than raw individual-level molecular data. The analytical pipeline began with the selection of the most strongly associated cis-QTL for each target gene, defined within a 1,000 kb window on either side of the gene, and only variants with a significant association (*p* < 5.0 × 10^−8^) were considered to ensure robustness. To mitigate potential biases due to allele frequency discrepancies across datasets, any SNP with an allele frequency difference greater than 0.2 between the LD reference, QTL, and GWAS datasets was excluded, following the default recommendation of the SMR software to minimize potential mismatches across datasets. Following this filtering step, we applied the HEIDI test, which distinguishes true pleiotropic effects from associations driven by LD. Associations with a P-HEIDI <0.01 were considered likely to reflect linkage and were removed from further analysis.

All SMR and HEIDI tests were conducted using the SMR software tool (version SMR v1.3.1), and only significant associations with a *p* < 0.05 and HEIDI test *p* > 0.01 were retained for subsequent colocalization analysis, for elucidating the potential causal link between genetically regulated methylation, expression, or protein abundance of mitochondrial genes and MDD (Wu et al., 2018).

The resulting set of associations provided candidate loci with potential causal effects on depression risk. These loci were then carried forward to colocalization analysis to further evaluate the likelihood that shared genetic variants underlie both the molecular trait and the disease phenotype.

### Colocalization analysis

To further evaluate whether the same genetic variant influences both a molecular trait and the risk of MDD, we conducted colocalization analysis using a Bayesian framework (Giambartolomei et al., 2014). The analysis was implemented using the R package coloc, which assesses five hypotheses via posterior probabilities (PPH): PPH0 (neither trait is associated), PPH1 (only gene expression is associated), PPH2 (only MDD is associated), PPH3 (both traits are associated but with distinct causal variants), and PPH4 (both traits are associated and share the same causal variant). In this study, a colocalization probability defined as [PPH4/(PPH3 + PPH4)] greater than 70%, a commonly used threshold in colocalization studies (Giambartolomei et al., 2014), was taken as evidence that, in the presence of a signal, the association signals, as supported by genetic instruments, for gene expression and MDD likely derive from a common causal variant.

To ensure both precision and biological relevance, the analysis was restricted to SNPs within a 1-Mb window around the target gene (from the transcription start to end sites), and additional quality control measures were applied to filter out low-quality variants. This approach not only minimizes confounding factors but also robustly identifies shared genetic determinants, thereby providing strong statistical support for a potential causal relationship between mitochondrial QTLs and MDD.

### Multi-omics association analysis

To systematically evaluate the strength of causal associations between mitochondrial-related genes across different regulatory layers (including methylation, transcription, and protein abundance) and MDD, we integrated multi-omics data using SMR analysis, supplemented by HEIDI testing to exclude confounding effects due to LD. We established a three-tier evidence grading system to rigorously filter putative causal genes based on the strength and consistency of associations across regulatory layers and datasets. Prioritization required significant protein QTL (pQTL) evidence, as proteins represent the final functional products of gene regulation and are more directly linked to cellular phenotype.

Tier 1: significant pQTL + concordant significant eQTL and mQTL in the same dataset, PPH4/(PPH3 + PPH4) > 0.70, and replication or consistent direction in an independent dataset. Tier 2: significant pQTL + significant and concordant eQTL or mQTL across two independent datasets, PPH4/(PPH3 + PPH4) > 0.70. Tier 3: significant pQTL plus two omics layers significant within one dataset, with consistent but not necessarily significant direction in another dataset, PPH4/(PPH3 + PPH4) > 0.70.

This grading system allowed us to systematically classify mitochondrial genes according to the strength of multilayered evidence. By integrating statistical associations, replication status, and biological concordance, we aimed to reduce false positives and identify high-confidence putative causal genes for future mechanistic studies.

## Results

### Integration of multi-omics evidence

Multilayer SMR identified five genes showing putative causal associations with MDD. These genes were prioritized based on the convergence of methylation, transcription, and protein-level associations, along with validation in independent datasets and evidence of colocalization.

*TDRKH* was prioritized as a Tier 1 gene. Methylation at a CpG site within this gene (cg24503712) was significantly associated with reduced gene expression and protein abundance, both of which were linked to a lower risk of MDD. These findings were replicated in the FinnGen dataset. The methylation, expression, and protein associations exhibited concordant directions of effect and passed both the colocalization threshold and HEIDI heterogeneity filter, supporting a robust causal relationship.

*METAP1D* was assigned Tier 2 status. Although no significant association was observed at the methylation level, both transcript and protein abundance showed consistent protective effects against MDD. These associations were validated in an independent dataset.

*LONP1*, *FIS1*, and *SCP2* were categorized as Tier 3 genes. Each showed protein-level associations with depression risk, accompanied by either methylation or gene expression associations within a single dataset. For *FIS1*, multiple CpG sites including cg17825709, cg25385322, cg01299997, and cg18158419 showed inverse relationships with expression, consistent with a protective effect. For *SCP2*, several CpG sites such as cg03609269, cg04310824, and cg12220753 were linked to reduced expression and elevated depression risk, while others such as cg00581603 and cg13078931 exhibited opposite trends, suggesting context-specific regulatory effects. *LONP1* demonstrated a similar bidirectional pattern, with sites like cg22499809 associated with increased risk and others such as cg00032366 and cg00815325 linked to protective effects.

**Figure 2.**
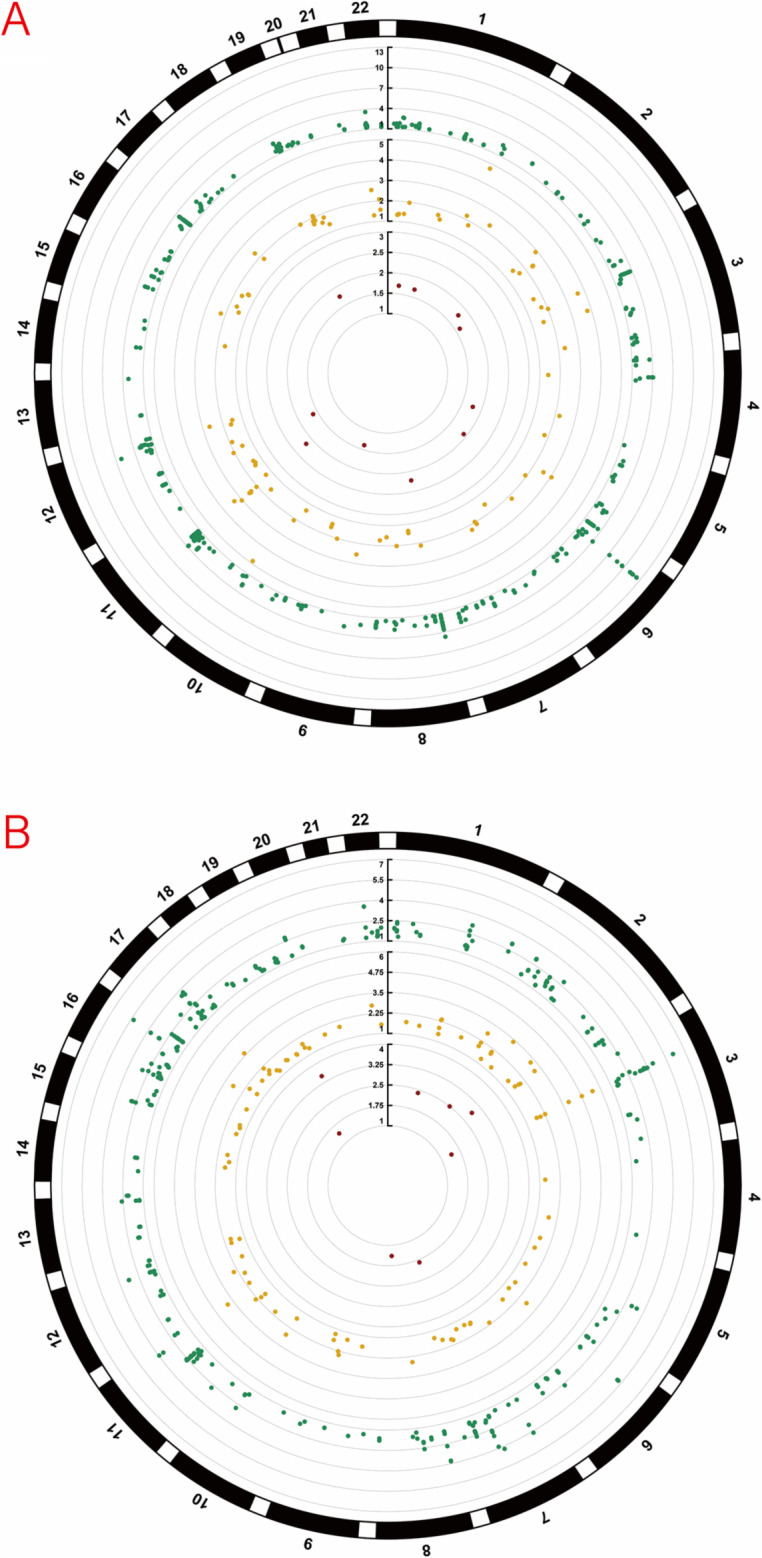
Circular Manhattan plots of multi-omics associations from two GWAS sources: (a) PGC; (b) FinnGen. Each circular plot displays three concentric rings: DNA methylation (mQTL, outer ring, green); gene expression (eQTL, middle ring, gold); and protein abundance (pQTL, inner ring, red). Dots represent loci associated with the respective traits, colored by chromosome. All loci shown meet nominal significance (*p* < 0.05). Genomic positions are arranged circularly, and the -log10(*p*) values are plotted radially for each ring.

**Figure 3.**
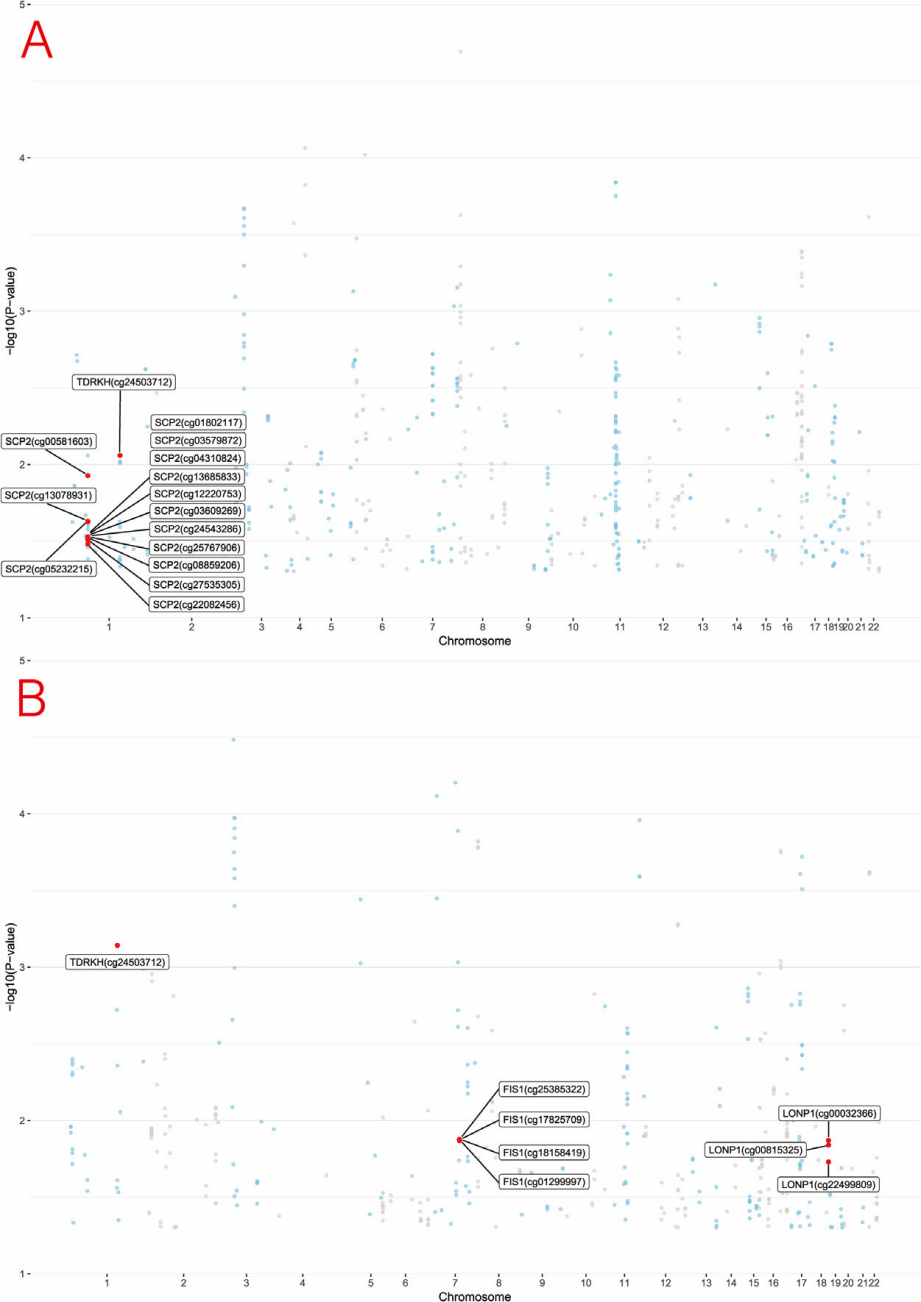
Manhattan plots of mQTL associations with MDD from PGC (a) and FinnGen (b). Each plot shows the genomic distribution of association signals across chromosomes. The –log₁₀(*p*) values represent the strength of association for loci mapped through mQTLs and integrated with eQTL and pQTL data. Chromosomes are alternately colored for visual clarity.

**Figure 4.**
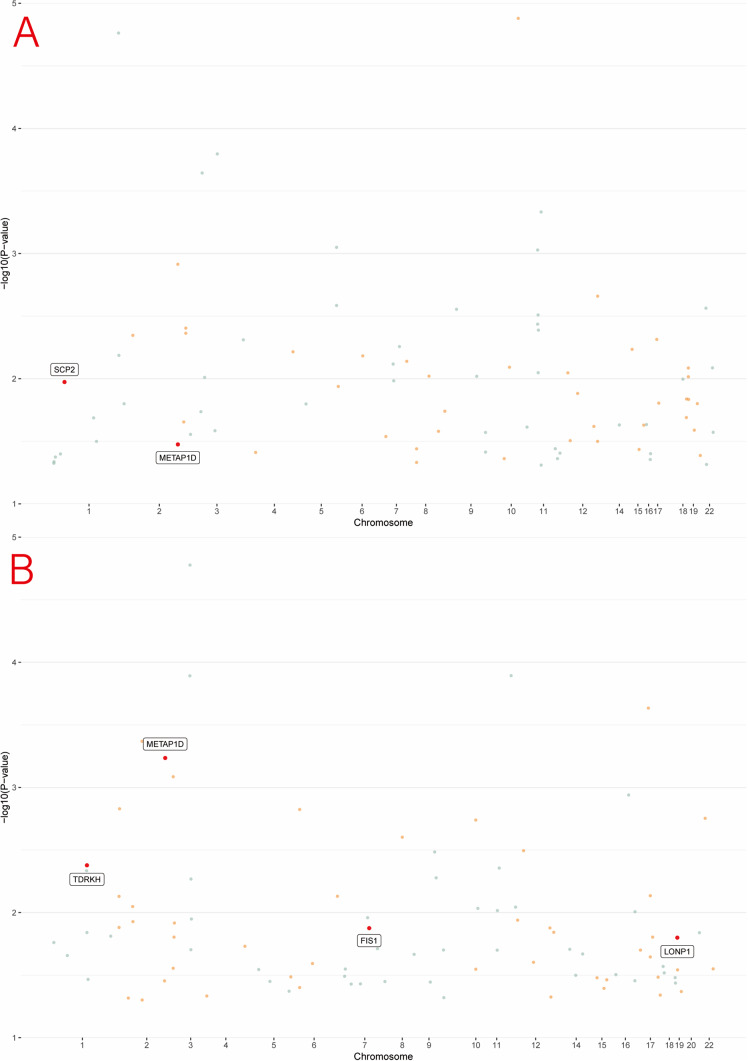
Manhattan plots of eQTL associations with MDD from PGC (a) and FinnGen (b).

**Figure 5.**
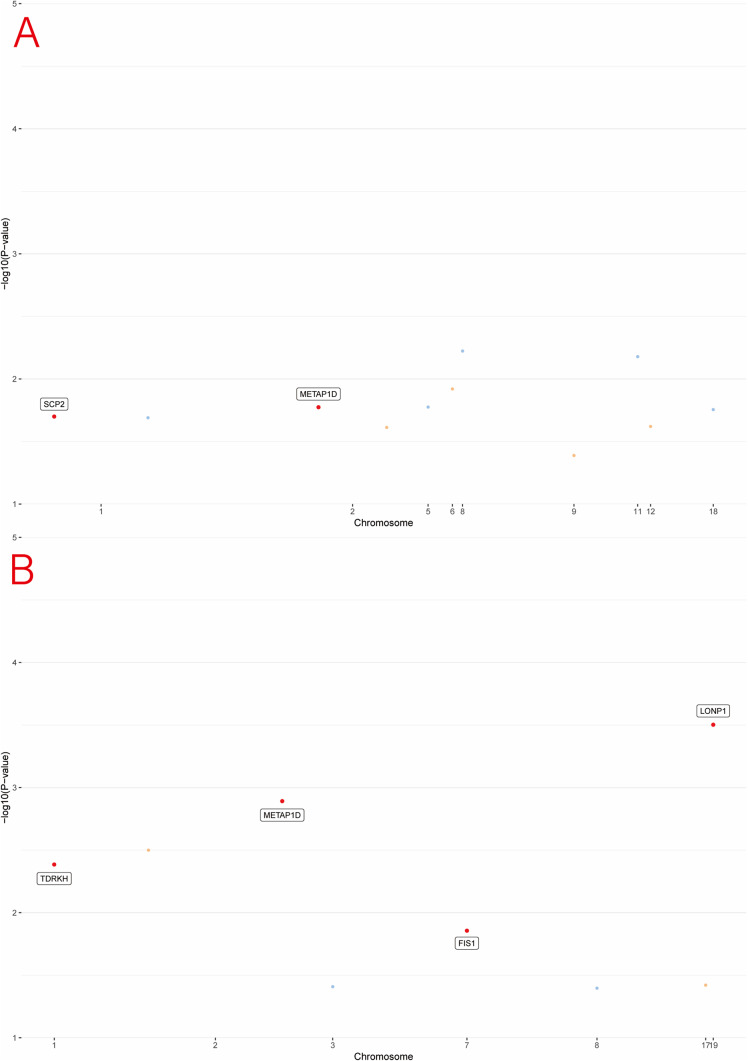
Manhattan plots of pQTL associations with MDD from PGC (a) and FinnGen (b).

Furthermore, the mQTL-eQTL and eQTL-pQTL associations for these genes were statistically significant and consistent with the observed regulatory patterns, while HEIDI analysis provided strong evidence of high concordance between these associations (P_HEIDI >0.01), thereby reinforcing the robustness of the identified causal relationships. Detailed results are provided in Supplementary Tables S8 and S9. A circular Manhattan plot illustrating the integrated multi-omics associations is shown in Figure 2. The corresponding Manhattan plots are presented in Figures 3, 4, and 5.

### Mitochondrial gene methylation and MDD

After removing P-HEIDI<0.01, 614 CpGs near 210 genes were observed (*p* < 0.05). Following FDR correction, 46 CpG sites located within regions of 20 unique genes were retained, among which 43 signals exhibited strong colocalization evidence (PPH4 > 0.70), supporting shared genetic regulation between DNA methylation and MDD.

Several genes displayed multiple CpG signals with divergent directions of effect. For *MSRA*, methylation at cg26621943 was associated with increased depression risk (OR = 1.10, 95% CI: 1.05–1.16). In contrast, methylation at cg26966828 and cg18786515 was associated with reduced risk (OR = 0.90, 95% CI: 0.85–0.96; OR = 0.93, 95% CI: 0.90–0.96, respectively). *SCP2* also demonstrated site-specific effects, with cg00581603 and cg13078931 associated with decreased risk, while other CpG sites in the same gene were linked to increased susceptibility. A similar bidirectional pattern was evident in *LONP1*, where methylation at cg22499809 increased depression risk, whereas cg00032366 and cg00815325 were associated with reduced risk.

**Figure 6.**
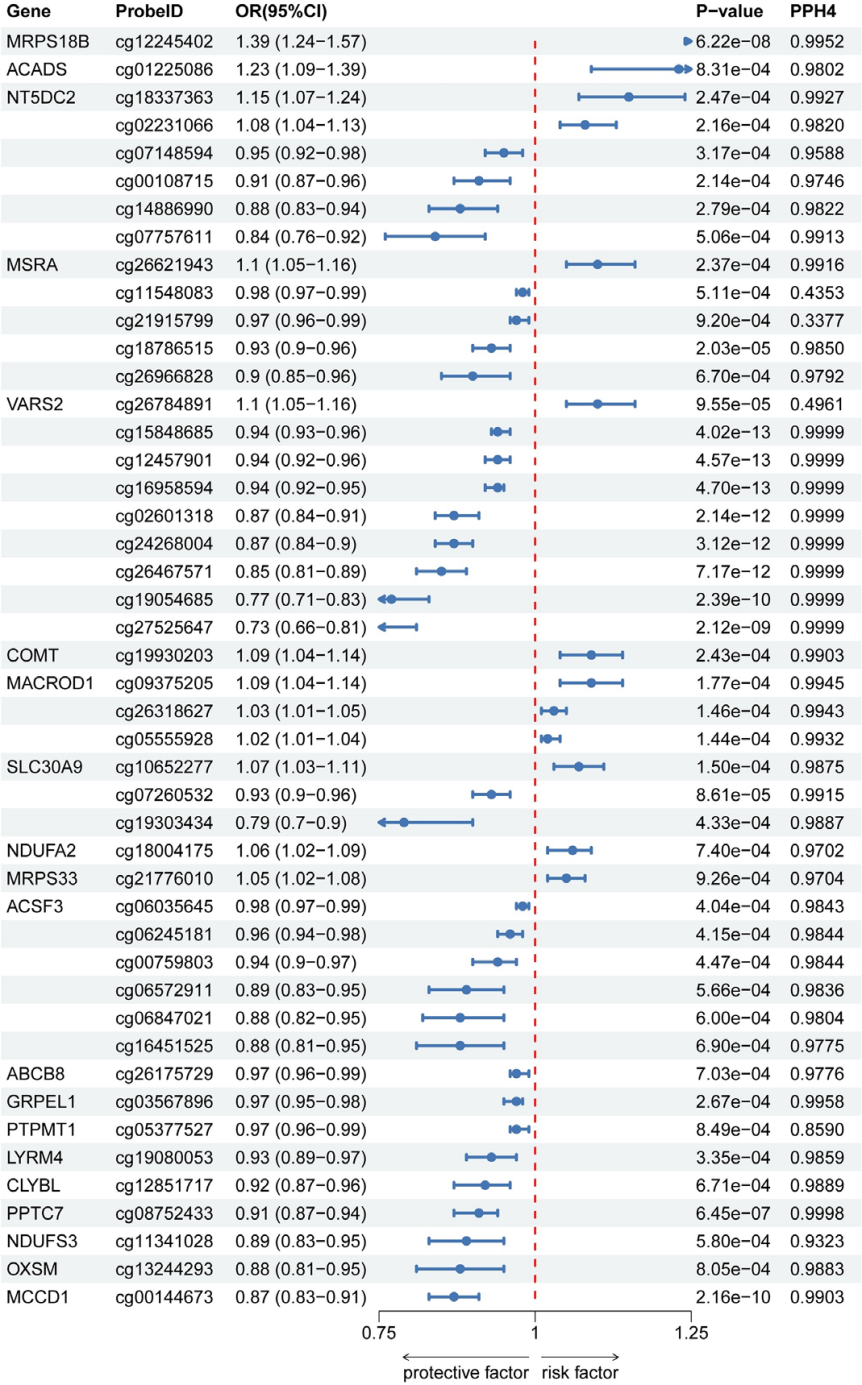
Associations of genetically predicted mitochondrial gene methylation with MDD in SMR (PGC). OR, odds ratio; CI, confidence interval; PPH4, Posterior Probability of Causal Variant.

**Figure 7.**
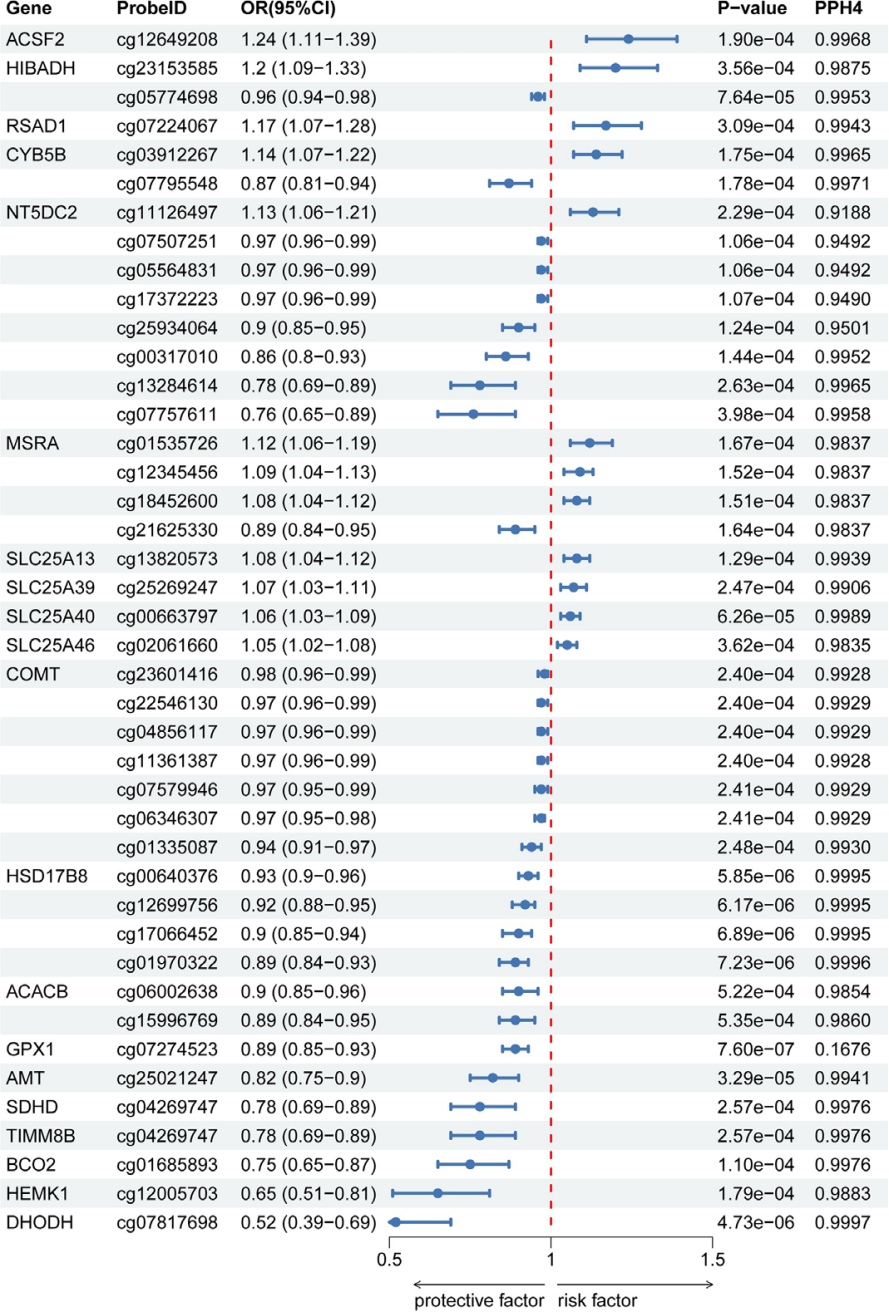
Associations of genetically predicted mitochondrial gene methylation with MDD in SMR (FinnGen). OR, odds ratio; CI, confidence interval; PPH4, Posterior Probability of Causal Variant.

Several methylation associations were replicated in the independent FinnGen dataset, reinforcing their robustness. Notably, the protective association for cg24503712 in *TDRKH*, previously identified in the integrative analysis, was validated in the replication cohort. The list of significant associated loci is provided in Supplementary Tables S2 and S3, with corresponding associations visualized in Figures 6 and 7.

### Mitochondrial gene expression and MDD

**Figure 8.**
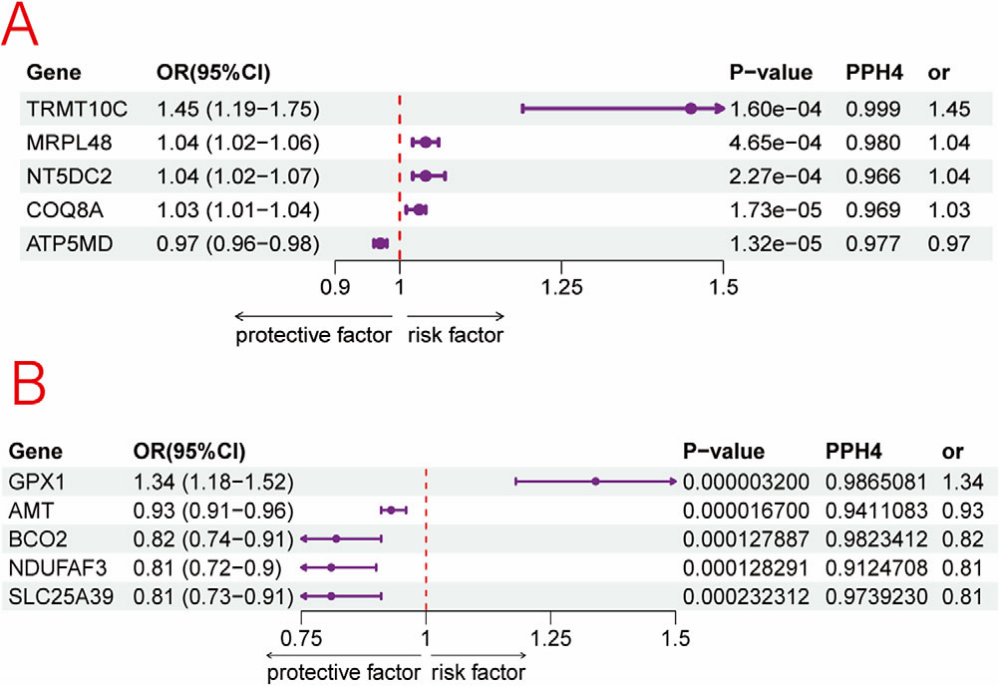
Associations of genetically predicted mitochondrial gene (eQTL) with MDD in SMR from PGC (a) and FinnGen (b).

After excluding associations with P-HEIDI <0.01, a total of 87 associations reached marginal significance. In the eQTL-SMR analysis, the expression levels of *TDRKH* and *FIS1* were positively correlated with MDD risk, while *METAP1D*, *SCP2*, and *LONP1* were associated with a reduced risk. Notably, the associations for *COQ8A*, *NT5DC2*, and *METAP1D* were replicated in the FinnGen database, further supporting the robustness of these signals. Significant eQTL results are summarized in Supplementary Tables S4 and S5, and their corresponding visual representation is shown in Figure 8.

**Figure 9.**
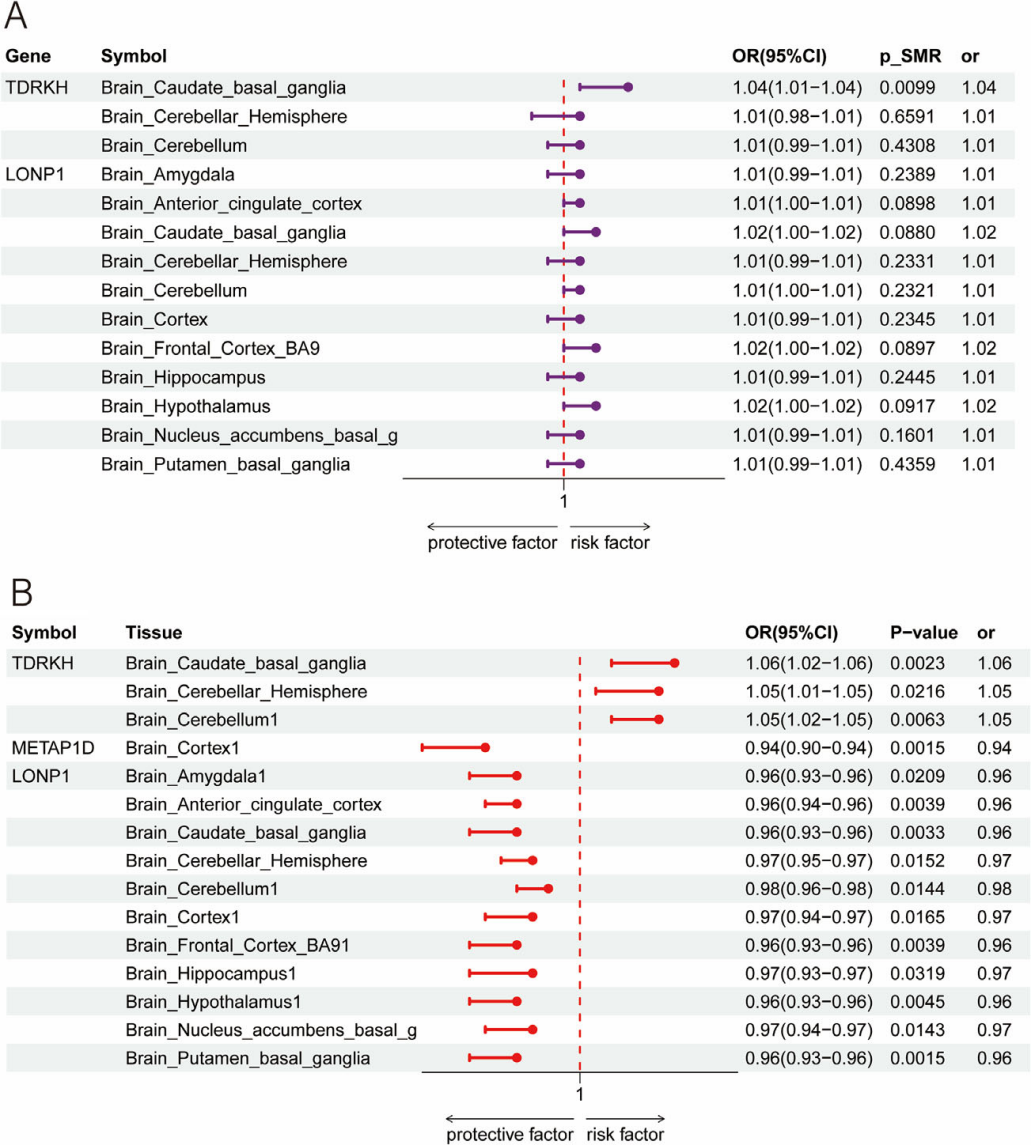
Brain eQTL forest plots for mitochondrial genes and MDD. (a) PGC: Associations between GTEx brain-region cis-eQTLs (exposures) and PGC MDD GWAS (outcome). The plot shows ORs and 95% CIs for mitochondrial genes, including *TDRKH* and *LONP1*, in the caudate basal ganglia, cerebellar hemisphere, cerebellum, and other brain regions. Significant associations are marked with *p* < 0.05. (b) FinnGen: Replication using the same GTEx brain eQTLs with FinnGen MDD GWAS as the outcome. ORs and 95% CIs for *TDRKH*, *METAP1D*, *LONP1*, and other genes across brain regions are displayed. Statistically significant associations (*p* < 0.05) are highlighted, confirming key findings from the PGC data.

To further explore the tissue relevance of the identified mitochondrial genes, we extended our analyses using brain-specific eQTL data from the GTEx Project as exposures and the PGC and FinnGen GWAS as outcomes. For *TDRKH*, significant associations with MDD were observed in the caudate basal ganglia when using GTEx eQTLs with both the PGC and FinnGen GWAS outcomes, with effect sizes in the range of OR = 1.04–1.06, while additional positive signals were consistently detected in the cerebellum and cerebellar hemisphere when using the FinnGen GWAS as the outcome, reinforcing the evidence from peripheral analyses. For *METAP1D*, no notable signals were observed when using GTEx brain eQTLs with the PGC GWAS, while when using the FinnGen GWAS, a protective association was identified in the cortex (OR = 0.94). *LONP1* showed consistent associations with MDD across multiple brain regions when using the FinnGen GWAS, including the amygdala, anterior cingulate cortex, and hippocampus, with odds ratios ranging between 0.96 and 0.98, while no significant effects were observed with PGC. No significant brain-specific associations were detected for *FIS1* or *SCP2* with either GWAS. Overall, the direction of these brain-based associations was concordant with the trends observed in blood-derived analyses (Figure 9 and Supplementary Tables S7).

### Mitochondrial proteins and MDD

**Figure 10.**
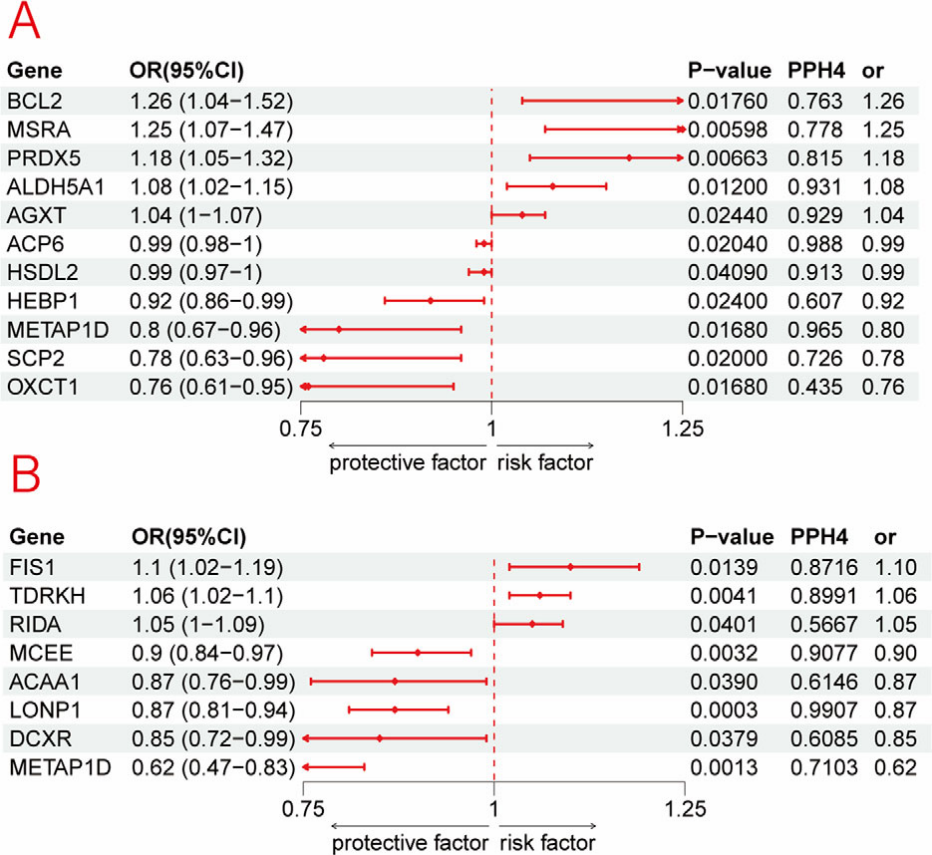
Associations of genetically predicted mitochondrial protein abundance with MDD in SMR from PGC (a) and FinnGen (b).

After excluding associations with P-HEIDI <0.01, and applying colocalization analysis (H4/(H3 + H4) > 0.70), we identified 11 mitochondrial proteins that were nominally associated with MDD risk (*p* < 0.05) in the PGC discovery dataset. Given sample size and the number of positive signals, multiple-testing correction was not performed. Significant pQTL–MR results are summarized in Supplementary Table S6 and visualized in Figure 10. At the protein level, genetically predicted abundances of *METAP1D* and *SCP2* showed inverse associations with MDD risk; for *METAP1D*, the direction of effect replicated in the FinnGen cohort, supporting robustness. In FinnGen, *LONP1* protein levels were negatively associated with MDD risk, whereas *TDRKH* and *FIS1* showed positive associations.

## Discussion

In this study, we systematically leveraged large-scale public GWAS datasets from the PGC and FinnGen cohorts, integrating MR, colocalization, and multi-omics analyses with MitoCarta3.0 (Rath et al., 2021) annotations to elucidate mitochondrial-associated genes potentially linked to MDD. Our results show that blood-derived methylation, expression, and protein signals strengthen the robustness of our gene prioritization and underscore their translational potential as accessible biomarkers for MDD. Our brain tissue results indicate that mitochondrial effects may exert brain-region–specific influences, particularly in the caudate, cortex, and cerebellum.

### Integration of multi-omics data to identify prioritized genes

We identified *TDRKH* as a prominent gene associated with MDD across multiple molecular levels, where hypomethylation at specific CpG sites was associated with increased gene expression and protein abundance, indicating an epigenetic-to-transcriptional-to-protein regulatory cascade. In the PGC discovery cohort, hypermethylation at cg24503712 was associated with a lower risk of MDD (mQTL OR = 0.96, 95% CI: 0.93–0.99), which replicated in FinnGen (OR = 0.93, 95% CI: 0.89–0.97); meanwhile, higher genetically predicted expression and protein abundance were associated with increased risk in FinnGen (eQTL OR = 1.04, 95% CI: 1.01–1.08; pQTL OR = 1.06, 95% CI: 1.02–1.10). In brain tissue analyses, *TDRKH* showed a significant positive association in the caudate when using GTEx eQTLs with the PGC GWAS (OR = 1.05, 95% CI: 1.01–1.10, *p* = 0.016) and FinnGen GWAS (OR = 1.04, 95% CI: 1.01–1.07, *p* = 0.009), with a nominal effect also observed in the cerebellum with FinnGen GWAS (OR = 1.03, 95% CI: 1.00–1.06, *p* = 0.048). This cross-layer pattern supports the plausibility that epigenetic variation may elevate transcription and propagate to protein-level changes, thereby contributing to MDD. *TDRKH*, located on the mitochondrial membrane, encodes a protein with Tudor and KH domains. Prior studies demonstrated that *TDRKH* serves as a mitochondrial anchor protein interacting with MIWI through its Tudor domain, catalyzing pre-piRNA trimming essential for piRNA stability and reproductive function (Izumi et al., 2016; Saxe, Chen, Zhao, & Lin, 2013; Wei et al., 2024). Furthermore, mutations in *TDRKH* have been linked to hereditary motor neuropathies (Dohrn & Saporta, 2020). Given that the KH domain is involved in RNA or single-stranded DNA binding (Lamb et al., 2000), *TDRKH* might regulate mitochondrial RNA metabolism. Our findings suggest a novel epigenetic role for *TDRKH* in MDD, warranting further validation.

Importantly, *METAP1D* showed a significant negative correlation with MDD across expression and protein levels, with consistent protective effects across cohorts. In PGC, increased expression and protein abundance were associated with reduced risk (eQTL OR = 0.92, 95% CI: 0.85–0.99; pQTL OR = 0.80, 95% CI: 0.67–0.96), with stronger protective effects in FinnGen (eQTL OR = 0.82, 95% CI: 0.73–0.92; pQTL OR = 0.62, 95% CI: 0.47–0.83). In brain cortex analyses, a protective association was observed with FinnGen GWAS (eQTL OR = 0.94, 95% CI: 0.90–0.99, *p* = 0.022), whereas analyses with PGC GWAS did not show a significant effect (eQTL OR = 0.98, 95% CI: 0.93–1.03, *p* = 0.41). This discrepancy may reflect differences in sample size and power across cohorts. *METAP1D* encodes a mitochondrial aminopeptidase localized within the mitochondrial matrix, responsible for removing N-terminal methionine from nascent proteins, and is critical for mitochondrial protein maturation and functionality (Cheng, Chi, Liang, Yu, & Wang, 2022; Lee, Kim et al., 2023). *METAP1D* emerges as a promising target in MDD, though brain replication is inconsistent.

### Complex regulatory patterns in multi-omics layers: LONP1, FIS1, and SCP2

*LONP1* exhibited complex, bidirectional methylation effects across CpG sites. Some CpG loci were linked to reduced MDD risk by aligning with protective downstream expression and protein changes, whereas others were associated with increased risk. In PGC, most associations were not significant. In FinnGen, cg22499809 was associated with increased MDD risk (OR = 1.08, 95% CI: 1.01–1.15, *p* = 0.028), while cg00032366 and cg00815325 showed protective effects (OR = 0.93, 95% CI: 0.88–0.99, *p* = 0.017; OR = 0.88, 95% CI: 0.79–0.97, *p* = 0.009). Beyond site-specific variation, *LONP1* demonstrated concordant protective associations across molecular layers in FinnGen (eQTL OR = 0.96, 95% CI: 0.93–0.99, *p* = 0.015; pQTL OR = 0.87, 95% CI: 0.81–0.94, *p* < 0.001), underscoring robust cross-layer evidence despite cohort-level heterogeneity. In brain eQTL analyses, protective associations were detected in multiple regions with FinnGen GWAS, including the amygdala (OR = 0.97, 95% CI: 0.94–1.00, *p* = 0.045), anterior cingulate cortex (OR = 0.96, 95% CI: 0.92–0.99, *p* = 0.012), hippocampus (OR = 0.96, 95% CI: 0.93–0.99, *p* = 0.018), and striatum (OR = 0.97, 95% CI: 0.94–0.99, *p* = 0.022), while no significant effects were observed with PGC GWAS.

Functionally, *LONP1* is an ATP-dependent protease located in the mitochondrial matrix that maintains proteostasis and cellular stress adaptation (Bahat et al., 2015; Lu et al., 2003). Impaired *LONP1* function leads to abnormal mitochondrial protein accumulation, oxidative damage, and subsequent neuronal dysfunction. Consistently, dysregulation of *LONP1* has been associated with abnormal CNS development (*Wang et al., 2019*) and neurodegenerative diseases, including Parkinson’s disease (Baden et al., 2023; C. Chen et al., 2023) and Alzheimer’s disease (Wang et al., 2023). Additionally, *LONP1* alleviates mitochondrial overload of active caspase-3 and HMGB1, thus preserving neuronal viability (Kim, Park, Kim, & Kang, 2021). These findings suggest a protective role of *LONP1* in MDD, supported by FinnGen.

For *FIS1*, we observed that methylation at several CpG sites was strongly associated with reduced gene expression. At the disease level, methylation instruments indicated lower MDD risk, whereas higher genetically predicted protein abundance was associated with increased risk (OR = 1.10, 95% CI: 1.02–1.19). Concordantly, genetically predicted expression showed a modest risk increase (OR = 1.04, 95% CI: 1.01–1.07) and was positively coupled with protein abundance, supporting a model whereby methylation-mediated downregulation of *FIS1* may be protective. In brain tissue analysis, no significant associations were detected with either PGC or FinnGen GWAS, suggesting that *FIS1* may have a limited role in brain tissue. *FIS1* localizes to the outer mitochondrial membrane and plays an important role in fission, biogenesis, remodeling, and quality control. Elevated *FIS1* expression has been shown to drive excessive mitochondrial fragmentation, impairing mitochondrial function and immune responses (Liu et al., 2023; Mukherjee et al., 2022). Moreover, *FIS1* regulates mitochondrial–ER contact sites, thereby modulating intracellular calcium dynamics and lipid exchange essential for neuronal survival. Dysregulation of *FIS1* may thus lead to mitochondrial dysfunction, calcium imbalance, and increased neuronal vulnerability, which could contribute to the pathogenesis of MDD (Ihenacho, Meacham, Harwig, Widlansky, & Hill, 2021), but the lack of brain replication suggests tissue dependence and need for validation.

In *SCP2*, multiple CpG sites exhibited heterogeneous methylation effects, with site-specific and bidirectional effects rather than a single monotonic pattern. Cross-layer analyses indicated nominally protective expression effects (eQTL OR = 0.98, 95% CI: 0.97–1.00) and a stronger protective signal at the protein level (pQTL OR = 0.78, 95% CI: 0.63–0.96). However, none of these associations were replicated in FinnGen blood analyses, and no significant associations were observed in brain tissues when using either cohort. Functionally, *SCP2* plays an important role in intracellular lipid transport, cholesterol homeostasis, and peroxisomal lipid metabolism (Dai et al., 2023; Galano, Venugopal, & Papadopoulos, 2022; Kriska, Pilat, Schmitt, & Girotti, 2010). Dysfunction in *SCP2* may disrupt neuronal lipid metabolism critical for membrane integrity, signal transduction, and synaptic plasticity, contributing to MDD susceptibility (Galano, Ezzat, & Papadopoulos, 2022; Horvath et al., 2015). Further, *SCP2* dysfunction may indirectly impact MDD risk through its associations with lipid metabolism disorders and neurodegenerative diseases frequently presenting with depressive symptoms (Bhatt, Nagappa, & Patil, 2020; Hong, Kim, & Im, 2016). Although blood-based analyses suggested a potential protective effect of *SCP2* in PGC, this finding did not replicate in FinnGen, and no significant associations were observed in brain tissues in either cohort. These discrepancies indicate that the role of *SCP2* in MDD remains uncertain and requires replication in larger cohorts.

## Limitations

First, eQTL datasets primarily derived from blood tissue restrict direct inferences regarding brain-specific mitochondrial regulation. Although we incorporated brain QTL data, limited sample sizes and bulk tissue heterogeneity likely contributed to inconsistent replication, particularly for *METAP1D* and *LONP1.* Second, while we combined HEIDI filtering and Bayesian colocalization to mitigate LD-driven false positives, these approaches reduce but do not fully exclude the possibility of horizontal pleiotropy. Third, our analyses were conducted in predominantly European populations, limiting generalizability to other ancestries. Finally, experimental validation remains absent, and our focus was limited to nuclear-encoded mitochondrial genes annotated in MitoCarta3.0, not mtDNA variation. Accordingly, mechanistic interpretations derived from these statistical associations should be regarded as hypotheses rather than established functional consequences. Future experimental studies will be essential to substantiate these findings, and future work integrating mtDNA variants, copy number, and haplogroup data alongside nuclear-encoded genes may provide a more comprehensive picture of mitochondrial contributions to MDD.

## Conclusion

Our multi-omics MR framework supports potential causal roles of mitochondrial genes in MDD. These findings underscore the importance of key mitochondrial genes, including *TDRKH*, *METAP1D*, *LONP1*, *FIS1*, and *SCP2*, offering valuable targets for future mechanistic research and therapeutic interventions.

## Supporting information

Liao et al. supplementary material Liao et al. supplementary material

## Data Availability

Data can be obtained upon a reasonable request to corresponding authors.

## Abbreviations

CI: confidence interval
CpG: cytosine-phosphate-guanine
DNA: deoxyribonucleic acid
eQTL: expression quantitative trait locus
FDR: false discovery rate
FinnGen: finnish biobank research project
GWAS: genome-wide association study
HEIDI: heterogeneity in dependent instruments
HMGB1: high-mobility group box 1
LD: linkage disequilibrium
MDD: major depressive disorder
MR: Mendelian randomization
mQTL: methylation quantitative trait locus
mtDNA: mitochondrial DNA
OR: odds ratio
PGC: psychiatric genomics consortium
pQTL: protein quantitative trait locus
PPH4: posterior probability of shared causal variant
QTL: quantitative trait locus
SNP: single nucleotide polymorphism
SMR: summary-based Mendelian randomization

## Supplementary material

The supplementary material for this article can be found at http://doi.org/10.1017/S0033291725102559.
